# Simultaneous Dual-Task Interventions That Improve Cognition in Older Adults: A Scoping Review of Implementation-Relevant Details

**DOI:** 10.1155/2022/6686910

**Published:** 2022-03-29

**Authors:** Natasha Versi, Kylie Murphy, Caroline Robinson, Mitchell Franklin

**Affiliations:** School of Allied Health Exercise and Sports Sciences, Charles Sturt University, Albury-Wodonga, NSW 2640, Australia

## Abstract

Cognitive change occurs as a person ages and may result in decreased cognitive functioning in older adults (60 years and older). Simultaneous dual-task (SDT) interventions—activities that require the person to engage physically and cognitively at the same time—are effective in improving cognition in this group. This scoping review analysed published implementation-relevant details about effective SDT interventions, to assist the translation of the available evidence into various practice needs and contexts. A total of 23 databases were searched for relevant studies published between 2009 and 2020 inclusively. Randomised controlled trials (RCTs) that revealed statistically significant improvement in one or more aspects of cognitive function were appraised for methodological quality, using the Joanna Briggs Institute RCT checklist. The 14 RCTs scoring over 50% on the checklist were further analysed. Implementation-relevant data—for example, intervention nature, session length and frequency, equipment and space requirements, target group, and outcomes—were extracted, collated, and synthesised. The effective SDT interventions varied considerably in their nature and outcomes, meaning that suitable SDT interventions exist for a range of contexts. However, the resources required to implement some of the interventions either were not reported and/or may be prohibitive in some real-world practice contexts. Whilst “challenge” and “group interaction” were common features of effective SDT interventions, most studies failed to report on key implementation details required to facilitate translation into practice. It also remains unclear for how long the cognitive benefits are sustained following an effective SDT intervention. In future, more consistent publication of information about how to implement effective SDT interventions, for whom these interventions are engaging/enjoyable, and why would help to translate the available research into improved cognitive outcomes for older adults.

## 1. Introduction

Cognitive change occurs as a person ages and may result in decreased cognitive function in older adults (aged 60 years and over). The term “cognitive” in this review refers to cerebral processes related to thinking. Although cognitive decline is not an inevitable outcome of ageing [1], when it does occur in one or more cognitive domains, a neurocognitive disorder (NCD) is said to be present [2]. The Diagnostic and Statistical Manual of Mental Disorders (DSM-5) identifies six cognitive domains that may be impacted: complex attention, executive function, learning and memory, language, perceptual and motor function, and social cognition [2]. According to the DSM-5, NCDs involve a disturbance in functionality in one or more of these cognitive domains, due to cerebral pathology [2]. NCDs are divided into two categories: mild and major [2]. The main distinction between mild and major neurocognitive disorder is the level of impact the impairment has on a person's ability to participate in daily activities [2]. The terms neurocognitive disorder and cognitive impairment are used interchangeably in the literature. The present review focused on neurocognitive disorder subtypes that are age-related and progressive (e.g., Alzheimer's disease, frontotemporal lobar degeneration, Lewy body disease, and vascular disease). Whilst the DSM-5 refers to “neurocognitive disorder,” the term “dementia” is still used widely in the literature and clinical practice. Therefore, these terms are used interchangeably in this review.

As the prevalence of cognitive decline increases with age [3], by 2050 it is predicted that the number of people living with a neurocognitive disorder could be around 100 million globally [4]. The Australian Institute of Health and Welfare [5] projected that Australian healthcare expenditure may increase by 364% from an estimated $3.85 billion in 2002-2003 to $17.84 billion in 2032-2033. The total cost of dementia is predicted to increase by 81% to $25.8 billion by 2036 [6]. The costs are predicted to further increase to $36.8 billion by 2056 which is a 2.6-fold increase in costs from 2016 [6]. The total costs include direct and indirect costs such as cost of hospitalisation, visits to health professionals, care, medication, and costs related to loss of productivity [6]. It is therefore a national and global health priority to address neurocognitive disorders proactively in order to minimise demands on healthcare systems.

In addition to the cost of neurological disorders on the healthcare system, there are significant personal impacts. Major neurocognitive disorders impact daily life significantly; for example, the person may forget appointments or how to cook a meal [7]. People with major neurocognitive disorders experience reduced autonomy and have generally been found to have a lower health-related quality of life compared to older people without known impairment [8]. People living with Alzheimer's disease have also been found to have substantially lower resilient coping mechanisms [9]. Implementing early interventions to delay the onset of neurological impairment and to maintain/improve functional capacity and independence for people living with a neurocognitive disorder is important for individuals, their families and communities, and health services.

In the last 10 years, a total of 11 systematic reviews and meta-analyses have focused on intervention studies in which at least one of the aims was to delay or slow cognitive decline related to age or cognitive decline associated with neurological disorders, through effortful and continuous physical and cognitive engagement [10–20]. The terms used in these reviews to describe an intervention that comprises both a physical and a cognitive element are inconsistent across the literature, including general dual-task, specific dual-task, dual-task, exergames, instrument playing, multicomponent, and separate interventions. Each cognitive and physical element of the intervention may be performed “sequentially” or “simultaneously.” Some of the systematic reviews differentiate between these two modes, and those which do tend to define “sequential” and/or “simultaneous” in uniformly consistent ways [11, 13–16, 18–20].

- “Sequential dual-task” interventions are defined as participation in a physical activity followed by a cognitive activity or vice versa.
- “Simultaneous dual-task” interventions are defined as participation in an activity that requires the person to engage physically and cognitively at the same time.

Systematic reviews consistently report that sequential and/or simultaneous interventions can be beneficial for improving cognitive function in older adults with and without cognitive impairment. A large majority of studies reviewed in the 11 systematic reviews report significant cognitive improvements after sequential and/or simultaneous intervention compared to either no intervention or an alternative intervention. These improvements have been observed in multiple cognitive domains.

However, systematic reviews that have focused on comparing the effectiveness of simultaneous versus sequential dual-task interventions have found that the effects of simultaneous dual-task interventions are larger than those of sequential dual-task interventions [11, 18]. For example, a review by Tait and colleagues [21] presented evidence supporting the superior effectiveness of simultaneous dual-task interventions in improving cognitive function in healthy and clinical populations. Examples of activities which result in cognitive improvements include dance, Tai Chi, exergames, karate, musical instrument playing, and activities such as memory training performed simultaneously with physical activities. The effectiveness of simultaneous dual-task interventions in delaying the onset, or slowing the rate, of cognitive decline can be explained by the concepts of neuroplasticity and cognitive reserve [22].

The intended purpose of the above-mentioned 11 systematic reviews was to investigate whether sequential and/or simultaneous dual-task interventions were effective. These reviews included broad information about the type of intervention, intervention duration, session frequency, study design and quality, and outcome measures. Whilst the information reported in these reviews is valuable, providing evidence supporting the effectiveness of dual-task interventions, other important information tends to be lacking. Details such as participant characteristics, steps involved in implementing the intervention, intervention setting, group size, participant engagement levels, training required to administer the activities, and other required resources (equipment, space, etc.) are either minimally described or not reported. Due to the lack of such details in existing reviews, it is difficult for health practitioners and other users to ascertain how and under what circumstances these interventions should and can be implemented.

In the current review, the focus was on the implementation of interventions which are classified as “simultaneous dual-task” (SDT) activities. Different forms of these activities will include some level of cognitive challenge performed simultaneously with physical activity but may also comprise physical activities which are inherently cognitively challenging, for example, learning a complex dance sequence or playing a musical instrument. The physical activity component of an SDT intervention may be a combination of activities, but the key point is the simultaneous combination of physical activity with cognitive challenge.

Despite the evidence supporting the effectiveness of SDT interventions, there are currently no published reviews that collate the implementation-relevant details reported in the respective studies. Such details are important for translating existing research into real-world practice. To address this gap in the current literature, the aim of this study was to systematically review the implementation-relevant details reported in published studies of SDT interventions shown to be effective in improving cognition in older adults. The purpose was not to evaluate the strength of the evidence, as this has already been done in numerous existing reviews, but instead to assist practitioners in (a) deciding which SDT interventions most suit their participant/client characteristics, goals, and settings, and (b) planning the implementation of relevant interventions. In summary, the aim was to assist those seeking to compare the benefits and requirements of evidence-based SDT interventions, to inform the selection and implementation of interventions most relevant to their needs and contexts.

## 2. Materials and Methods

### 2.1. Overall Design

Scoping reviews are classified by the Joanna Briggs Institute (JBI) as a type of systematic review [23]. Whereas systematic reviews typically aim to answer questions related to the feasibility, appropriateness, meaningfulness, or effectiveness of a certain treatment or practice, scoping reviews are focused on the identification of certain characteristics or concepts in studies and then mapping, reporting, or discussing these characteristics [23, 24]. As systematic reviews that report on the effectiveness of SDT interventions in improving cognitive outcomes for older people have already been published [11, 18], the aim of the present study was to collate implementation-relevant details of the SDT activities that have been found to be effective. Therefore, a scoping review was appropriate. The PRISMA-ScR checklist was used to ensure that the conduct and reporting was rigorous and transparent [25].

### 2.2. Information Sources

The following 23 databases were searched for articles published between January 2009 to December 2020 inclusively: Scopus; EBSCOhost (Health); ProQuest Health and Medicine Database; Health Collection; MEDLINE ALL (1946 to present); Pedro; OTseeker; speechBITE; PsycBITE; JBI COnNECT on OVID; JBI COnNECT; eTG: Electronic Therapeutic Guidelines; Cochrane Library; EBM Reviews; Web of Science Core Collection; ScienceDirect; Consumer Health Complete; Sage Journals; Wiley Online Library; Springer Link; PubMed Central; AusportMed; and Google Scholar.

### 2.3. Search

The JBI three-step search strategy was used to ensure a comprehensive review [23], conducted by the principal investigator (first author). As per the first step in the JBI three-step search strategy, an initial search was conducted using the primary databases in November 2019. EBSCOhost, PubMed, Scopus, and Cochrane Reviews were the primary host servers. Key terms are not well established because this area of research is relatively new and, therefore, research articles use variations of terms to describe activities that incorporate both physical movements and cognitive stimulation within the same activity (SDT interventions). Therefore, the following combinations of terms were used when searching these databases: “simultaneous activit^∗^” OR dual-task^∗^ OR “challenging activit^∗^” OR (cognitive AND physical) AND “older people OR elderly OR older adult^∗^ OR senior OR age^∗^” AND “cogniti^∗^ OR dementia OR neuro^∗^” to ensure that all possible alternative words/phrases were captured.

As per the second step, the key terms found in the relevant articles from the primary databases were applied to the more extensive list of databases identified earlier. Apart from the search terms identified above, two more terms were noticed in studies which described simultaneous dual-task activities. These terms were “mental^*∗*^ stimulat^*∗*^” (which captured, for example, mentally stimulating) and “cogniti^*∗*^ stimulat^*∗*^.” These additional terms were added to the original search terms. The final search syntax was applied to all 23 databases.

Within the constraints of the respective databases, the following tools were used: “English language only” limiter, “studies published from January 2009 onwards” limiter, and the multifield option, and the search syntax was applied to the titles and abstracts only. The recommended articles were viewed, and the reference lists of all the articles that met the inclusion criteria were manually reviewed, to ensure that all relevant articles were captured as per the third step of the JBI process [23].

### 2.4. Eligibility Criteria and Selection of Sources of Evidence

**Figure 1:**
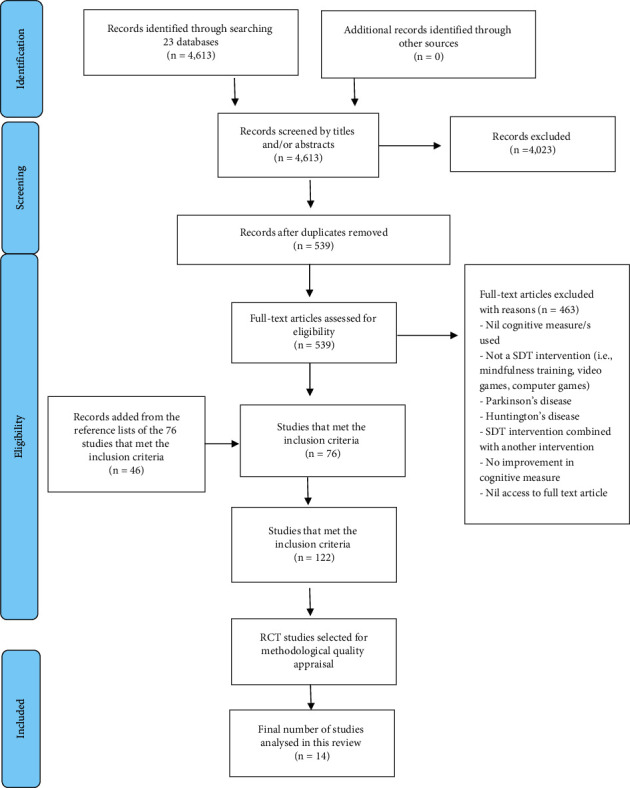
PRISMA flowchart.

During the article identification and screening process, all articles regardless of study design and methodological quality were included. It was a requirement for this review that the study must have reported a statistically significant improvement in at least one cognitive measure, or in brain structure, following an SDT intervention. All participants regardless of original cognitive level—no cognitive impairment, mild cognitive impairment, or dementia—were included. As depicted in Figure 1, numerous studies were initially identified, but qualitative studies were excluded because they did not satisfy the criterion of “statistically significant improvement.” Articles written in languages other than English, papers that could not be accessed as full text, and studies that did not include at least one cognitive measure or brain scan were also excluded. To remain within the scope of this study, studies of older adults with cognitive changes related to reasons other than ageing and/or dementia (such as Parkinson's disease and Huntington's disease) were excluded.

According to the National Health and Medical Research Council, randomised controlled trials (RCTs) are the second highest level of evidence, following systematic reviews of randomised controlled trials [26]. Given the number of RCTs that were found, it was decided that only RCTs would be included in the next step of the review process.

### 2.5. Critical Appraisal of the Sources of Evidence

This scoping review is based upon the assumption that SDT activities are effective; only studies where interventions were found to be effective were therefore of interest. Consequently, there was a minimum standard of methodological quality required to ensure a level of confidence that the intervention was indeed effective. In alignment with the “study selection stage” in Peters and colleagues' framework [23], RCTs that met the inclusion criteria underwent quality appraisal to determine the overall methodological quality. Not all scoping reviews include this step [23]; however, critical appraisal is important to establish internal validity and therefore trustworthiness of the included studies.

To efficiently and effectively determine the methodological quality of the studies, the RCT JBI critical appraisal checklist was used [27]. The principal investigator (first author) executed quality appraisal of 41 RCTs and determined the overall quality score for each individual article. A second reviewer (MF) cross-checked the scores of six randomly selected RCTs using the same JBI RCT checklist. Therefore, in total, 68 questions for six RTCs were compared. The second reviewer initially gave a different answer to four of the 68 questions but after discussion agreed that it was appropriate to align the answers with the original assessment. The very high level of agreement confirmed the reliability of the principal investigator's scoring. The 14 RCTs which scored over 50% on the checklist were included in the final analysis [28–41]. The remaining RCTs which scored below 50% were excluded from further analysis. Figure 1 shows a modified PRISMA-ScR flowchart [25] which summarises the above-described steps and outcomes.

### 2.6. Data Charting Process, Data Items, and Synthesis of Results

Details about the SDT interventions in the 14 RCTs were tabulated. The following details were extracted: methodological quality (calculated score); inclusion criteria; exclusion criteria; sample characteristics; intervention description; country; setting; facilitator details; group size; session length and frequency; total duration; cognitive measures; assessment procedures; assessment timing; reported *p* values and effect sizes; original health status (medical status, mobility, functioning, falls history, and frailty); training characteristics (training intensity, cognitive difficulty); space and equipment needs and other costs of carrying out the activities; attrition rates; and participant motivation data. This information was initially typed into a Microsoft Excel spreadsheet. Details not reported in the articles were recorded as unreported. All the extracted data were ultimately clustered into five thematic areas and transferred, in summary form, to five tables to facilitate recognition of patterns and tendencies.

## 3. Results

Table 1 presents the overall quality appraisal scores and indicates the reporting transparency in relation to how the cognitive measures were implemented. Overall, the methodological quality of included studies was low. Using the JBI checklist as a guide, most of the studies did not explicitly state the following: whether the participants, interventionists, and assessors were blinded to group allocation/treatment assignment; analysis of differences between completers and non-completers; and detailed descriptions of how outcome measures were assessed in a reliable way. The study by Li and colleagues [33] had the highest (76.9%) appraisal score. Overall, less than half (5/14) of the studies reported on how all the assessments were implemented, but 40% of the studies reported that all the assessments were completed in an electronic format. The three studies [28, 29, 35] that used a method of neuroimaging or neuroplasticity assessment to measure neuronal changes after SDT intervention, reported adequately on the assessment techniques. Reliability and validity of the cognitive measures used were not reported by most studies (11/14). Less than half (6/14) of the studies did not also report on who administered the cognitive measures.

Table 2 shows that a range of cognitive functions were measured. Supplementary materials are included to provide further information on the cognitive measures and brain scans that showed improvement in the included studies. Due to the inclusion criteria for this study, all reviewed interventions resulted in improvement between and/or within groups on at least one measure. “Between groups” refers to differences between intervention and control groups; “within group” refers to pre-to-postintervention changes within a group. Three of the fourteen studies measured neuronal changes including neuroplasticity assessment [28], a functional near-infrared spectroscopy test to measure prefrontal cortex activity during walking (fNIRS treadmill) [29], and magnetic resonance imaging (MRI) scans to determine whole brain volume [35]. All of these three studies demonstrated positive brain changes following the SDT intervention. Only just over half (8/14) of the studies reported calculated effect sizes. At least one large effect size was reached for the majority (6/8) of these studies, in multiple cognitive domains. The effectiveness timeframe varied significantly between studies; for example, cognitive improvements were observed after one session of exergaming [33] and after 6 months of Tai Chi Quan exercise [33] and music-based multitask exercises [31]. Most (11/14) studies assessed cognition immediately before and after intervention only. Outcomes were measured for a period after the cessation of the intervention in only three of the studies [35, 37, 41]. Two of these studies reported sustained, statistically significant cognitive improvements 2–5 months after completion of the intervention [35, 37]. Several (8/14) studies did not report if there were any adverse outcomes during and/or after the intervention. One study reported falls during the classes [33], and in one study a participant with post-polio syndrome experienced leg pain whilst doing the activity [38]. No other adverse outcomes were reported in any of the studies reviewed.

Table 3 shows how the SDT interventions were implemented. The reported types of interventions included exergames (4/14); Tai Chi (3/14); dance/movement training (1/14); music-based multitask exercise classes (1/14); net step exercise (1/14); Qigong exercises (1/14); theatre-based exercises (1/14); and other tasks requiring simultaneous cognitive and physical engagement (2/14). However, half (7/14) of the studies did not report the specific movements, games, or exercises at all, and two of the 14 studies reported only a few examples of the tasks performed in the intervention. Almost half (6/14) of the studies reported increasing levels of challenge/intensity in the exercises; one study reported low intensity [28]; and the remaining seven studies did not report on the level of challenge or difficulty at all. Group size, session length, and frequency varied significantly between studies. The mean session length was a little over 51 minutes; the average frequency was two sessions per week; and the average number of total sessions was 31. In over half (8/14) of the studies, interventions were conducted exclusively in pairs or groups. A majority of articles reported on what equipment was used (10/14 studies) and the facilitator details (10/14 studies). Most of the interventions were facilitated by professionals with expertise in the relevant activity, trained students, or trained volunteers (9/14 studies). Only one of the studies reported the exact amount of space required to perform the intervention [30], and most (11/14) did not report on costs associated with the intervention. Many studies (9/14) reported on the venue, but visual diagrams of the intervention set-up or movements were rarely presented.

Table 4 shows the types of participants the interventions were effective for. The participants were all over the age of 60 with BMI ranging from 23.9 to 29.2 (kg/m^2^). The participants were able to participate in the respective interventions despite varying cognitive and physical capability, health, and risk status and living situation. Most of the participants were cognitively intact—defined as no sign of dementia or cognitive impairment [30–32, 35, 37, 39]. Two studies involved participants with mild cognitive impairment [34, 40]; two studies included participants that were cognitively intact and had mild cognitive impairment [28, 38]; and two studies included participants with dementia [33, 41]. Many (8/14) studies did not report on the participants' living situation. However, in studies which did report this variable, participants were living in either independent living centres/units/apartments, long-term care, residential care facilities, or day centres. Many (8/14) studies did not report on whether the participants experienced falls nor the frailty of participants.

Table 5 shows the measures of engagement reported in the articles. One study reported high training enjoyment levels among the participants during a dance exergame, through applying the German eight-item version of the physical activity enjoyment scale [29]. Overall, the studies reported high adherence rates ranging from 68.1% to 100% (where adherence is defined as the extent to which the participants participated in the activity) and variable attrition rates ranging from 0% [32, 34, 37, 39] to 47.8% [30].

## 4. Discussion

The purpose of this scoping review was to collate implementation-relevant details of simultaneous dual-task (SDT) interventions shown to be effective in improving cognition for older people, to facilitate translation into practice. Following a comprehensive search for relevant articles and critical appraisal, 14 RCT articles were included in this review. Details relevant to choosing and implementing SDT interventions in healthcare practice contexts were extracted, summarised, tabulated, and synthesised.

### 4.1. Key Findings

This review has revealed several important findings. A wide range of SDT interventions have been studied. Most studied interventions require significant resources or training, but some are potentially accessible to many people/organisations and could be modified to suit a range of participants' abilities. Sustained cognitive benefits were rarely investigated but warrant future research. An appropriate level of challenge appeared to be important in achieving the cognitive gains reported in the reviewed articles; social interaction may also be significant. In general, the level of reporting transparency in relation to the implementation of effective SDT interventions is problematically low, hindering translation into practice. Finally, there was very little reporting about participant motivation or enjoyment during the interventions which, along with practical implementation details, is information that would be of interest to many practitioners.

### 4.2. The Interventions Varied Widely

A range of effective intervention types were studied in the reviewed articles, for example, exergames, Tai Chi, and music-based exercises. Frequencies and durations also ranged widely, from one 30- to 45-minute session [34] to two 60-minute sessions per week for 6 months [33]. This variability in effective interventions can be viewed as encouraging for primary healthcare practitioners and community members. The wide variety of effective SDT interventions provides practitioners with options to present and discuss with participants/clients to meet the client's preferences and goals, which enhances the practicality of these interventions. Additionally, interventions were studied for a range of older adults with and without cognitive impairment. Although effective SDT activities are available to suit people with different capabilities, it is important to consider whether a specific SDT intervention is culturally appropriate for an identified group and whether the activities can be implemented in a wide range of contexts. Particular SDT interventions can also be tailored; for example, fewer Tai Chi movements can be chosen for older people with dementia to memorise and follow. A few interventions such as Tai Chi and dance can be implemented in different formats, such as individually, in a group setting, in virtual groups, outdoors, or through online videos. This means that people of all ages and abilities, with varying resources and contexts, can participate in preventative SDT activities to delay or slow cognitive decline. These findings are significant for not only practitioners working with older adults but also people of all ages living in the community, who could incorporate these activities within their daily routines.

### 4.3. Most Studied Interventions Required Significant Resources or Training

Many interventions required resources and/or training that might be considered prohibitive for some community health and aged care settings, but some effective interventions are more accessible (e.g., Tai Chi and dance). The resources needed to implement a specific activity are dependent on the type and format (e.g., individual versus group tasks). One category of effective interventions is exergames, which are technology-driven physical games [28]. Exergames require specialised equipment such as stationary recumbent bikes, dance platforms, step mania software, video game consoles, Wii fit plus, and EA sports active games. Therefore, the purchase of exergame equipment might make this type of SDT intervention difficult to implement in a primary healthcare context; training in how to operate the exergame technology may also be required. Other interventions such as dance/movement training, music-based multitask exercises, net step exercise, and simultaneous cognitive and physical tasks require props such as visual images, exercise balls, piano, Fumanet®, and Octaband®. These props may not be readily available in some community and aged care settings. Additionally, these activities require a certified facilitator or trained volunteers to provide structure and guidance as to how to perform the activities. Tai Chi based interventions, however, require less overall resourcing as compared to other activities because they involve the participants memorising and/or following Tai Chi movements from the Tai Chi master. Therefore, it appears that most of the studied interventions have an associated cost due to the requirement of special resources and/or facilitators. This means that not all healthcare professionals/aged care workers/older people living in the community would be able to implement these SDT interventions, as not all settings/contexts have such resources readily available. Therefore, future research investigating the effectiveness of low-cost, low-skill but high quality SDT interventions is needed to facilitate wider-spread implementation of and engagement in SDT activities.

### 4.4. Sustained Benefits Are Possible

A vital implementation detail to consider is how long the cognitive benefits persist before the person needs to reengage in the activity to maintain or enhance cognitive performance. Only three studies reported on follow-up effects, but two out of those three studies did report sustained cognitive benefits (2 to 5 months after intervention) [35, 37]. However, the third study did not find a sustained effect at follow-up [41]. These findings suggest that cognitive benefits may be evident for up to five months after completion of the SDT intervention. It is unknown whether cognitive benefits persist beyond five months as none of the reviewed studies followed up with participants beyond this timeframe. Therefore, further research is required to understand how frequently activities/boosters are required after intervention, to maintain the positive benefits. Determining the sustainability of these SDT interventions would also provide valuable information in relation to the long-term costs and resources required for implementation. Indeed, other reviews investigating the effectiveness of dual-task activities also highlight the limited number of studies reporting follow-up outcomes [11–14]. Determining the sustainability of benefits is important, particularly when considering costs versus outcomes.

### 4.5. Role of Progressively Increasing Cognitive Challenge

One common theme in the reviewed interventions was the idea of “challenge.” Challenge is likely to play a role in the effectiveness of SDT interventions. Whilst only some articles explicitly reported that the intervention involved challenge (including progressively increasing challenge), the other interventions may have been experienced by the participants as challenging if the activities were novel to them. However, the reviewed studies did not specifically measure the level of challenge involved.

Existing evidence indicates that exercising in an environment which has aspects of cognitive challenge is necessary to induce neurologic effects such as neuroplasticity and cognitive reserve [15, 18]. One experimental study [42] has specifically investigated high demand (cognitively challenging) and low demand (non-cognitively challenging) activities and their effects on cognition in older people; this study found that novel high demand activities enhanced memory function in older adults. As SDT interventions combine cognitive engagement/stimulation and physical movements, a sense of cognitive challenge is likely involved, and this is likely to produce beneficial neurological changes in the brain. Additionally, dual-task interventions are known to decrease bilateral prefrontal cortex oxygenation, which is associated with improved efficiency of executive functions [20]. During high-intensity physical activities, reduced oxygenation in the prefrontal cortex indicates that cognitive resources are being redirected to maintaining motor function [29, 43]. All three studies in the present review that measured brain changes after activity reported increased brain volume, increased brain-derived neurotrophic factors (BDNF), or decreased prefrontal oxygenation. In other words, SDT activities with an aspect of cognitive challenge may delay or slow cognitive decline by simultaneously promoting neuroplasticity and cognitive reserve and decreasing prefrontal cortex oxygenation. From an implementation perspective, it is important that an appropriate level of physical and cognitive challenge is achieved to ensure that the person capitalises on the neurological and cognitive benefits. Future research is required to determine how “challenging” an activity needs to be to maximise neurological benefits, taking account of the participant's preintervention status and abilities. Future research should also explore assessment tools/methods that can be used to measure the level of challenge.

### 4.6. Possible Role of Social Interaction and Peer Support

It is important to consider the degree to which factors such as social interaction and peer support might have contributed to the improved cognitive outcomes associated with the SDT interventions. Many studies reported that the intervention had a group element or was performed in pairs. The success of interventions that were facilitated in a group format suggests that social interaction and peer support may have contributed to the level of engagement and motivation of the participants during the activities. Additionally, the high number of studies conducted in a group format also suggests that conducting these activities in groups, as compared to conducting them individually, may be a factor contributing to improved cognition and positive neurological changes in the brain. It is important to consider these factors as they affect how SDT interventions might be best implemented to optimise the benefits. A meta-analysis conducted by Burke and colleagues [44] found that physical activity interventions conducted in “true groups” was superior to standard exercise classes with minimal group interaction. The authors defined true groups as interventions where group dynamics were deliberately promoted [44]. Two of the reviewed studies reported that an “encouraging atmosphere” or “noncompetitive atmosphere” was promoted [31, 35], and one study reported that competition was promoted [28]. The level of competitiveness and support from other participants could have also contributed to an individual's motivation to continue to engage in the intervention; this should be further explored in future research.

### 4.7. Need for Better Reporting of Implementation-Relevant Details

Implementation of specific evidence-based interventions by practitioners and the wider public is the ultimate aim, but this is hampered when limited implementation-relevant and contextual details are provided in the publications that report on these interventions. Many studies did not report on the specific movements/exergames/exercises associated with the intervention, how the equipment/props were used, costs, adverse outcomes, risk status, or the living situation of participants. These details are critical for the translation of interventions into practice; studies that do not report on these key details provide limited guidance for implementation. Considering that many different SDT interventions are effective in improving cognitive function, the reason for not utilising an intervention should be related to client or clinician-related factors, rather than a lack of implementation details. Interventions such as Tai Chi and dance appear to be the most easily transferable to everyday situations, in contrast to other novel activities such as music-based and theatre-based exercises which require specific details to enable practitioners to replicate these interventions effectively. Many of the SDT interventions did not provide visual diagrams of the activity set-up, which would be helpful for practitioners to facilitate implementation. The potential for adverse outcomes is also crucial information for practitioners who might implement these interventions, given that older adults who perform two tasks at the same time are at a heightened risk of falls [20].

### 4.8. Need for Better Reporting about Participant Engagement

Two of the studies reported high attrition rates for participants of 30–50% [30, 38]. There could be a range of factors that influenced almost half of the participants in one study to drop out of the intervention [30], but the reason for attrition was not stated. Other than attrition and adherence, almost all studies failed to report on any aspect of participant motivation (e.g., enjoyment). Future studies should include measures of participant motivation and report information that explains participant attrition, as this would assist a practitioner's decision making about the appropriateness of an intervention for a specific client group. A clearer understanding of the reasons for “dropout” would help to facilitate engagement and continuing participation in effective activities, to produce long-term cognitive benefits.

### 4.9. Directions for Future Research

This review contributes to the field of implementation science and serves to highlight a gap between research and knowledge translation, resulting from a lack of implementation details in published literature. The findings of this review highlight the importance of future research investigating SDT interventions, to increase awareness of the value of these activities and how to best implement them. Given the resource-intensive nature of many of the interventions that have been studied to date, valuable information could be gained from future research focused on interventions that are more affordable and accessible in various contexts. This research should include explicit reporting about the level of challenge involved for participants and effectiveness comparisons between interventions involving varying levels of challenge. Future research should also involve follow-up assessments to ascertain for how long the effects are sustained following an intervention. Most importantly, future studies should also report on all the key implementation details to enable translation into practice. To increase participation in SDT activities, future research investigating the barriers, enablers, and motivators for participation in such activities by different population groups would also be valuable. Incorporating subjective measures of engagement (e.g., motivation) would be helpful from a clinician's perspective, to inform clinical reasoning when choosing interventions for their client groups. Additionally, other factors such as social interaction (including competition) and peer support should be investigated further to understand their impacts on participation, adherence, and attrition. As improved cognition positively impacts other aspects of life such as increased social participation [45], future studies exploring secondary benefits such as quality of life would expand knowledge and may promote implementation of SDT activities in a range of contexts.

### 4.10. Limitations of This Review

There are several limitations of this review to acknowledge. Firstly, it is important to highlight that this scoping review focused only on improved cognitive/brain measures. Additionally, this review only included articles written in English, which may have limited the transferability of the findings to Western contexts. Articles that could not be accessed in full were excluded as well. Only studies that reported effectiveness of SDT interventions were reviewed; numerous studies that did not show improvement were excluded. Moreover, the results presented in this review relate to SDT groups only, not comparison-activity groups; the comparison activities in some cases were as effective as the SDT activities. Studies that may have been published in activity-specific journals (e.g., music-based journals) may have been missed, as mainly health databases were searched. Intervention studies published since 2019 were also not included. Additionally, the study identification, screening, and eligibility stages of the review process could have been more rigorous, with a second reviewer cross-checking every article during each stage to increase inter-rater reliability. Finally, it was beyond the purpose of this review to conduct a comparative analysis regarding the relative effectiveness of the different kinds of intervention. Therefore, no conclusion can be drawn from this review regarding whether specific types of SDT activities are more effective than others.

## 5. Conclusion

This review has found that SDT interventions take many forms and can be modified to suit different ability levels and situational contexts. Published studies show that SDT interventions are effective in improving cognition for older adults; however, a lack of reported implementation details is a barrier to translation into a practice setting. Hence, future research should report on all key implementation details to enable the application of effective interventions by both practitioners who work with older adults and individuals who seek to maximise and prolong their cognitive function into older adulthood.

## Figures and Tables

**Table 1:** Methodological quality and reporting transparency of the included articles.

Study	Methodological quality score	Summary of cognitive assessment implementation
[28] Anderson-Hanley et al.	61.5%	Brain-derived neurotrophic levels for the neuroplasticity assessment analysed using enzyme linked immunosorbent assay. Method of implementing cognitive measures not reported
[29] Eggenberger et al.	53.8%	5 paper-and-pencil cognitive tasks. FNIRS treadmill task adequately analysed and describe
[30] Esmail et al.	53.8%	Individually assessed in a quiet room. Executive function tests assessed using 3 tablet tasks on an iPad Air 16GB using Safari as the browser. The Montreal cognitive assessment administered as a paper-and-pencil task.
[31] Hars et al.	53.8%	Not reported
[32] Kitazawa et al.	69.2%	Computer based assessments. Touch-M system running on the Microsoft Windows operation system and a touch-panel type desktop PC
[33] Li et al.	76.9%	Not reported
[34] Monteiro-Junior et al.	61.5%	Not reported
[35] Mortimer et al.	53.8%	MRI acquisition and analysis reported. Method of implementing other cognitive measures not reported
[36] Noice and Noice	61.5%	Not reported
[37] Norouzi et al.	53.8%	Computer version of the *N*-back test
[38] Schoene et al.	69.2%	Stroop stepping test; two stance panels of the step pad used. Method of implementing cognitive measures not reported
[39] Silsupadol et al.	61.5%	Auditory Stroop and counting backwards by threes, performed in a seated position
[40] Sungkarat et al.	61.5%	Not reported
[41] Tsang et al.	61.5%	Not reported

All included studies were randomised controlled trials (RCTs).

**Table 2:** Ways in which the studied interventions were effective (*p* < 0.05).

Study	Intervention type	Cognitive domains assessed (as stated in individual studies)	Cognitive measures that improved statistically significantly (within and between group differences)	Effect sizes (within and between group differences)	Effectiveness timeframe	Reported adverse outcomes
[28] Anderson-Hanley et al.	Cybercycle exergame group	Executive function Visuospatial function Attention Verbal fluency Verbal memory Neuroplasticity	Between groups: colour trails, digit span backward, neuroplasticity assessment and Stroop C. Within group: colour trails, digit span backward, neuroplasticity assessment and Stroop C	Between groups: (*η*_*p*_^2^) large effect size: colour trails test, digit span backward and Stroop C. Within group: (d) medium effect size: colour trails, digit span backward, Stroop C, and reduction in risk of clinical progression to mild cognitive impairment	Cognitive measures: baseline and after intervention (3 months). Neuroplasticity assessment: immediately before and after intervention	Not reported
[29] Eggenberger et al.	Dance exergame	Information processing Shifting attention Inhibition Working memory General cognitive ability	Between groups: fNIRS treadmill test: reduced *HbO*_2_ at preferred walking speed in the left and right prefrontal cortex (PFC). At preferred and fast walking speed, higher left PFC activity than right. *HbO*_2_ levels higher at fast compared to preferred walking speed. Cognitive measures: unclear. Within group: unclear	Between groups: (r) medium effect sizes: fNIRS treadmill test. Within groups: (r) unclear	Immediately before and after intervention (8 weeks)	Nil adverse outcomes
[30] Esmail et al.	Dance/movement training	Executive function Nonexecutive function Global cognition	Within group: *N*-back test, digit Stroop and dual-task tests	Within group: (*ηp*^2^) large effect size: *N*-back test, digit Stroop test, and dual-task tests	Immediately before and after intervention (12 weeks)	Nil adverse outcomes
[31] Hars et al.	Music-based multitask exercise classes	General cognition Executive functioning	Between groups: sensitivity to interference subtest of the frontal assessment battery. Within group: Mini-Mental State Examination	Not reported	Immediately before and after intervention (6 months)	Not reported
[32] Kitazawa et al.	Net step exercise	Visuospatial function. Level of cognitive decline	Between groups: Touch-M, accuracy of the order of a process and naming fingers subtests of the touch-panel type dementia assessment scale. Within group: Touch-M total score and naming fingers subtest of the touch-panel type dementia assessment scale	Not reported	Immediately before and after intervention (2 months)	Not reported
[33] Li et al.	Tai Chi Quan exercise	Global cognition	Between 2 intervention groups and stretching control group: Montreal cognitive assessment	Within group: improvement on Montreal cognitive assessment scores; it is unclear if statistical significance was reached	Not reported	Immediately before and after intervention (6 months)
[34] Monteiro-Junior et al.	Exergame Wii group	Semantic memory Short-term memory Working memory	Within group: verbal fluency test	Within group: (Δ before and after) large effect size: verbal fluency and digit span forward tests. Small effect size: digit span backward test. Between groups (Δ%): large effect size: verbal fluency, digit span forward and backward tests	Immediately before and after intervention (after 1 session)	Not reported
[35] Mortimer et al.	Tai Chi group	Not reported	Between 2 intervention groups and control group: increased whole brain volume. Within group: category verbal fluency test, auditory verbal learning test (delayed recognition), rail making test part A and B, Mattis dementia rating scale total score and attention, initiation and memory subtests of the Mattis dementia rating scale	Not reported	Before intervention and at follow-up (40 weeks). 19-20 weeks between conclusion of intervention and follow-up assessment	Not reported
[36] Noice and Noice	Theatre group	Word recall Category fluency Episodic memory Problem solving	Experiment 1 Between group: means-end problem solving procedure, east Boston memory, verbal fluency, and Word list recall tests. Within group: means-end problem solving procedure. Experiment 2 Between group: means-end problem solving procedure, east Boston memory, verbal fluency and word list recall tests. Within group: means-end problem solving procedure, verbal fluency, and word list recall (immediate) tests	Experiment 1 Between groups (*η*_p_^2^): large effect size: means-end problem solving procedure, east Boston memory, verbal fluency, and word list recall tests. Within group (*η*_p_^2^): medium effect size: means-end problem solving procedure; small effect size: verbal fluency. Experiment 2 Between group (*η*_p_^2^): large effect size: means-end problem solving procedure, east Boston memory, verbal fluency, and word list recall tests. Within group (*η*_p_^2^): large effect size: means-end problem solving procedure, verbal fluency, and word list recall (immediate) tests	Immediately before and after intervention (4 weeks)	Not reported
[37] Norouzi et al.	Motor-cognition dual-task group	Working memory	Between groups: *N*-back test. Within group: *N*-back test	Large effect size: *N*-back test, after test and at follow-up. Between groups: unclear *η*_p_^2^ or d; large effect size: *N*-back test, after test and at follow-up	Before intervention, after intervention, and at follow-up (4 weeks), 8 weeks between conclusion of intervention and follow-up assessment	Not reported
[38] Schoene et al.	Exergame group	Combined stepping and executive function Processing speed Executive function Attention Inhibition Visuospatial function	Between groups: letter digit test, hand reaction and choice stepping reaction times, mental rotation, and executive subtest of the attentional network test. Within group: difference between low and high adherers: letter digit, alerting subtest of the attentional network test and trail making test B/A	Not reported	Before and after intervention (16 weeks)	One participant with post-polio syndrome reporting severe leg pain. No other adverse effects reported.
[39] Silsupadol et al.	Simultaneous balance and cognitive task group	Not reported	Between groups: counting backwards by threes and auditory Stroop tests whilst sitting. Within group: counting backwards by threes and auditory Stroop tests whilst sitting	Within group: (*η*_p_^2^) large effect size: counting backwards by threes and auditory Stroop tests. Between groups: (*η*_p_^2^) large effect size: counting backwards by threes and auditory Stroop tests	Before and after intervention (4 weeks)	Not reported
[40] Sungkarat et al.	Tai Chi group	Episodic memory Visuospatial function Executive function Hand reaction time	Between group: logical memory-delayed recall subtest of the Wechsler memory scale, block design, trail making test B-A and hand reaction time. Within group: not reported	Not reported	Before and after intervention (15 weeks)	No adverse effects
[41] Tsang et al.	Qigong exercise group	Global cognition	Between groups: acute and mid intervention effects; thinking operations subtest of the LOTCA-G. Within group: not reported	Between groups: (*η*_p_^2^) small effect size: acute affects; orientation, visuomotor organisation, thinking operations, memory and attention subtests of the LOTCA-G	Acute effects: baseline to after intervention (12 weeks). Mid-intervention effects: baseline to mid intervention (6 weeks). Maintenance effects: post- to follow-up assessment (20 weeks)	No adverse events

The within group data relates to the SDT group only.

**Table 3:** How the interventions were implemented.

Study	Intervention type	Intervention summary details	Group size	Session length and frequency (min/week)	Total sessions (n)	Training format	Equipment	Space required	Facilitator details	Costs	Venue type
[28] Anderson-Hanley et al.	Cybercycle exergame group	3D tours via a virtual reality display on the recumbent bike. Competition promoted. Details of the specific 3D tours played not reported. Link was provided to appendixes; however irrelevant webpage appears. Not reporting if rides were challenging. Low intensity	38	Gradually increased ∼45 min × 3	Min 25 rides	Not reported	Indoor stationary recumbent bikes	Adequate space for the bikes	Not reported	Not reported	8 independent living facilities
[29] Eggenberger et al.	Dance exergame	Learnt different stepping sequences via an interactive video game. Specific exergames played not reported. Progressively difficult stepping patterns	22	30/3x	24	2 participants practised in exergame at the same time using the same screen	StepMania software installed on a screen. Ropes hanging from above to maintain balance. Two 1 × 1 m impact dance platforms with 4 pressure sensitive areas, positioned side by side	Adequate space to perform exergame	2 trained postgraduate students led groups of 4	Not reported	Geriatrische Klinik (geriatric clinic)
[30] Esmail et al.	Dance/movement training	Different themes explored through expressive movements and guided gestures. How themes were explored in sessions and specific movements not reported. Challenge or training intensity not reported	23	60/3x	36	Group format of 4–8 participants	Props including Octaband, CoOper Blanket, Elastablast, colourful scarves, exercise balls, TheraBand & exercise balls. Music to accompany sessions	Space of ∼4.5 m × 10 m	Registered dance movement therapist or a supervised trainee, with additional training	Not reported	Gym facility at a geriatric institution research centre
[31] Hars et al.	Music-based multitask exercise classes	Different multitask exercises executed to the rhythm of piano music. Few exercises identified. Type of music and rhythmic patterns not reported. The difficulty of exercises progressively increased	66	60/1x	25	Exercises performed individually or in groups	Piano music objects	Adequate space for sessions	Not reported	Not reported	Not reported
[32] Kitazawa et al.	Net step exercise	Memorised and practiced different net step exercises with and without singing. Schedule of net step exercises detailed in appendix of study. Encouraging atmosphere promoted. Increasingly difficult net step designs	30	60/1x	8	Group format of ∼10 people. One participant practised at a time, whilst others were seated around the Fumanet	One 4 × 1.5 m Fumanet net comprised of 50 cm × 50 cm squares arranged in a 3 × 8 grid. Columns labelled A, B, C	Rows numbered 1–8. Every square assigned a coordinate. Illustrations of exercises provided to instructors	Adequate space for group sessions	10 volunteers given 6 hr lectures on exercise and received net step exercise accreditation. Following a protocol	Transport provided for all sessions & assessments
[33] Li et al.	Tai Chi Quan exercise	Learnt 8 modified Tai Chi Quan forms with built-in variations and integrated therapeutic movement exercises. Specific exercises described in detail in the protocol. Increased intensity of exercises	224	60 min/ 2x	48	Group format of 9–21 participants in each session	Not reported	Adequate space for group sessions	Not reported	Costs for all study expenses: $202,949 ($906 per person)	15 class sites at community centres, churches, or nonprofit organisation in 7 cities in Oregon
[34] Monteiro-Junior et al.	Exergame Wii group	6 games performed with virtual feedback. Reporting specific selected games. Specific movements associated with the games not reported. Challenge or training intensity not reported	9	30–45/1x	1	Not reported	All 6 games selected from Wii fit plus and EA sports active games	Adequate space to perform movements	Supervised by sports medicine professional or physiotherapist	Not reported	Not reported
[35] Mortimer et al.	Tai Chi group	Warmup, Tai Chi, and cooldown exercises. Specific Tai Chi exercises not reported. Challenge or training intensity not reported	30	50/3x	120	Group format	Not reported	Open space to practise Tai Chi	1 TC master & assistant	Not reported	Jing an park or gymnasium
[36] Noice and Noice	Theatre group	Specific increasingly demanding theatrical exercises; nature not reported. No memorisation of lines. Held scripts during rehearsal and performance. Noncompetitive atmosphere. The specific exercises involved in experiments 1 and 2 are not reported	Exp 1: 38 Exp 2: 17	70/2x	8	Group format	Scripts	Adequate space for group sessions	Experiment 1: experienced assistant activity director Experiment 2: 1 experienced acting teacher. E-mail descriptions, calls, and in person conversations prior to each class	$40 for completion of all sessions All 3 homes similar in cost	3 retirement homes
[37] Norouzi et al.	Motor-cognition dual-task group	Simultaneous resistance training plus 12 cognitive tasks. Specific tasks reported. Challenge or training intensity not reported	20	60–80/3x	12	Not reported	Isokinetic exercise device. Props: cards, visual images, word lists	Adequate space for sessions	Trained clinical physiotherapists supervised resistance training	Not reported	Not reported
[38] Schoene et al.	Exergame group	4 multilevel games played through an interactive training system consisting of a step pad interacting with video game technology. Specific games played and how the step pad interacted for each game reported. Encouraging to progress to higher levels to ensure games were sufficiently challenging	39	20/3x	48	Individually played	Interactive training system. Home television screens required for installation	Adequate living room space to play the 4 games	Unsupervised. Individually instructed on how to operate the system in a 90 min session. Phone calls made and home visits offered if requested	Not reported	The 4 games were played on standard television screens in the homes of the participants
[39] Silsupadol et al.	Balance and cognitive tasks	Examples of 3 cognitive and balance tasks reported. Equal focus to balance and cognitive tasks. Challenge or training intensity not reported	8	45/3x	12	Individualised sessions	Not reported	Adequate space to perform exercises	Not reported	Not reported	Not reported
[40] Sungkarat et al.	Tai Chi group	Warm up, 10-form Tai Chi principles, and cool down exercises. Specific movements practised in classes and in the 50 min video not reported. Challenge or training intensity not reported	33	50/3x	TC classes: 9 TC exercises at home: 36	Group format of 6-7 participants for TC classes and individual TC exercises at home	50 min TC video Logbook	Adequate space for group TC classes. Adequate space at home to complete TC home exercises	Classes led by certified Tai Chi instructor. Unsupervised Tai Chi home exercises. 50 min video provided to complete Tai Chi home exercises	Not reported	Weeks 1-3: classes held in exercise room at department of physical therapy. Weeks 4-15: practising Tai Chi at home.
[41] Tsang et al.	Qigong exercise group	10 qigong movements completed 2/3 times each session in sitting or standing styles. Program development reported. Movements and breathing techniques reported. Intensity not reported	69	60/2x	24	Group format	Not reported	Adequate space for group sessions	5 qualified Qigong instructors	Not reported	Not reported

**Table 4:** Types of participants in the interventions.

Study	Intervention type	Age (years)	Average BMI (kg/m^2^)	Health status	Original functioning (physical activity levels, mobility, preexisting routines, and everyday functioning in daily activities)	Original cognitive status	Living situation	Risk status (falls and frailty)
[28] Anderson-Hanley et al.	Cybercycle exergame group	≥75	29	No neurological conditions and written physician approval	Adequate cybercycle functionality Average kcal/day reported	42% with mild cognitive impairment diagnosis	Independent living centres	Not reported
[29] Eggenberger et al.	Dance exergame	>65	24.4	No recent head injury and consent from physician for chronic/terminal illnesses	Ability to walk for approximately 10 min on a treadmill. Average treadmill walking speed reported	No Alzheimer's disease/dementia diagnosis	Independent or senior residence facilities	Not reported
[30] Esmail et al.	Dance/movement training	>60	Not reported	Range of medications reported. No known conditions; no hormone therapy, smokers, or general anaesthetic taken recently. Some reported family history of cognitive disorder. Good hearing and vision reported	<150 min/week of moderate intensity and structured exercise. No mobility limitations or neuropsychologically tested. ≤2 standard measures of alcohol pre day reported. Ability to complete ADLs and IADLs	Cognitively intact. Mini-Mental score of ≤24 were excluded	Not reported	Some reported falls in the last year. Not frail
[31] Hars et al.	Music-based multitask exercise classes	>65	26	Some reported having psychotropic medications; prostheses; tendon rupture; vision problems; low comorbidities; cardiac, nervous system, and balance disorders	Average kcal/week and Timed Up and Go assessment scores reported	Cognitively intact Mini-Mental score >25	Not reported	Some reported falls in the last year. Physically frail
[32] Kitazawa et al.	Net step exercise	>70	Not reported	No severe heart failure, dementia, cognitive and physical conditions	Self-reported ability to walk independently >10 min without sitting or using assistive devices. No net step exercise and ability to drive prior to intervention. Timed Up and Go assessment score reported	Cognitively intact	Not reported	Not reported
[33] Li et al.	Tai Chi Quan exercise	>70	29.2	No major medical and physical conditions. Some reported having chronic conditions and taking multiple medications. Approval from practitioner to participate in intervention	Impaired mobility but ability to walk 1/2 blocks with or without assistive devices. Timed Up and Go assessment score reported. No daily/structured physical activity, >15 min walking, or muscle strengthening activities done recently	Dementia Mini-Mental score ≤20	Not reported	All had at least one fall recently
[34] Monteiro-Junior et al.	Exergame Wii group	>65	66	Some reported taking multiple medications. No cardiovascular conditions, acute musculoskeletal injury, delirium but some reported having diseases	Independent ambulation	Mild cognitive impairment. Ability to communicate. Mini-Mental score between 22 & 25	Long-term care	Not reported
[35] Mortimer et al.	Tai Chi group	>60	Not reported	No stroke, neurological conditions, contradictions for MRI. Some reported being smokers	No participation in vigorous exercise or Tai Chi practice. Excluded if unable to walk unassisted for 2 km or maintain balance. Average time spent exercising reported	Cognitively intact. No cognitive impairment/dementia. Mini-Mental score >26	Not reported	Not reported
[36] Noice and Noice	Theatre group	>68	Not reported	Some reported taking multiple medications. No conditions affecting participation reported. Ability to hear instructions and read large type scripts	Ability to stand and move about for few minutes with or without assistive devices. Sufficient everyday functioning to participate in theatre exercises	Ability to comprehend instructions and execute movements to communicate meaning of the scene	Independent living sections or assisted living units	Not reported
[37] Norouzi et al.	Motor-cognition Dual-task group	≥65	Not reported	No lower extremity injuries, myocardial infarction, uncontrolled blood pressure, Alzheimer's disease, visual impairment, mental and neurological conditions	Ability to stand upright for ≥2 min and walk 20 m without auxiliary equipment. Good physical activity status. Physical activity levels reported	Cognitively intact. Mini-Mental score ≥ 24	Not reported	Not reported
[38] Schoene et al.	Exergame group	>70	Not reported	Some reported taking multiple medications. No major lower extremity pain, corrected vision of less than 6/16, colour-blindness, cognitive, neurocognitive and unstable conditions. Low functional comorbidity reported	Ability to walk with or without a walking aid and step unassisted on a step pad	Cognitively intact and mild cognitive impairment. Mini-Cog score >3	Independent living apartments of retirement villages	Some reported falls and concerns about falling
[39] Silsupadol et al.	Simultaneous motor and cognitive dual-task group	≥65	Not reported	No neurological and musculoskeletal diagnosis reported. All reported having balance impairment	Berg balance scale average score of <52 and/or walking with a self-selected gait speed of ≤1.1 m/s included	Cognitively intact Mini-Mental score >24	Not reported	Not reported
[40] Sungkarat et al.	Tai Chi group	>65	23.9	Some reported taking multiple medications. No neurological, depressive symptoms, acute & chronic conditions, or drugs taken for cognition	Physical ability to participate in intervention. Excluding individuals who engaged in regular exercise ≤30 min/day, ≤3 days per week	Amnestic multiple-domain mild cognitive impairment. Adequate memory and ability to comprehend instructions. Mini-Mental score >26	Not reported	Some reported falls in the last year
[41] Tsang et al.	Qigong exercise group	>80	Not reported	Not reported	Able to walk with or without assistive devices	Mild dementia. Mini-Mental score >23	Residential care facilities or day centres	Frail

The data relates to the SDT group only.

**Table 5:** Indicators of engagement in the interventions.

Study	Intervention type	Attrition (%)	Adherence (%)	Motivation/enjoyment level
[28] Anderson-Hanley et al.	Cybercycle exergame group	21	78.95	Not reported
[29] Eggenberger et al.	Dance exergame	13.6	91.4	High training enjoyment rating
[30] Esmail et al.	Dance/movement training	47.8	90.74	Not reported
[31] Hars et al.	Music-based multitask exercise classes	15	79	Not reported
[32] Kitazawa et al.	Net step exercise	0	100	Not reported
[33] Li et al.	Tai Chi Quan exercise	13.4	86.61	Not reported
[34] Monteiro-Junior et al.	Exergame Wii group	0	100	Not reported
[35] Mortimer et al.	Tai Chi group	0.03	96.7	Not reported
[36] Noice and Noice	Theatre group	Experiment 1: 14 Experiment 2: 10.53	Experiment 1: 86 Experiment 2: 89.47	Not reported
[37] Norouzi et al.	Motor-cognition dual-task group	0	100	Not reported
[38] Schoene et al.	Exergame group	31.9	68.1	Not reported
[39] Silsupadol et al.	Simultaneous dual-task group	0	100	Not reported
[40] Sungkarat et al.	Tai Chi group	10.6	89.4	Not reported
[41] Tsang et al.	Qigong exercise group	11.5	88.5	Not reported

These values are only representative of the SDT group.

## Data Availability

The data extracted from the reviewed studies have been listed in the references. No additional data from previously published articles were used in this scoping review.

## Supplementary Materials

Supplementary Materials The cognitive measures that improved in the included studies.
