# Supplementary material for: Simultaneous Dual-Task Interventions That Improve Cognition in Older Adults: A Scoping Review of Implementation-Relevant Details
Source: J Aging Res. 2022 Mar 29;2022:6686910. doi: 10.1155/2022/6686910 (PMC8983262; doi:10.1155/2022/6686910)
Supplement: Supplementary Materials — The cognitive measures that improved in the included studies. [file 6686910.f1.docx]

**Cognitive measures that improved in included studies**

| **Measure** | **Cognitive domain/s** | **Brief summary description** | **Psychometric properties** |
| --- | --- | --- | --- |
| 1-back task  2-back task | Working memory | A sequence of visual stimuli presented. Participants recall stimuli that match current stimulus (Gajewski et al., 2018). | Weak concurrent validity (Jaeggi et al., 2010)  Good construct validity (Kearney-Ramos et al., 2014)  Mixed reliability of the online N back test (Kulikowski & Potasz-Kulikowska, 2016) |
| Digit Stroop  Stroop C  Auditory Stroop task | Inhibition  Processing speed  Attention  Cognitive flexibility  Working memory | Identifying colour of ink/pitch of sound/number of visual stimuli of contrasting word/digit whilst ignoring the meaning of the word/digit and vice versa (Esmail et al., 2020; Anderson-Hanley., 2012; Silsupadol et al., 2009). | Adequate test-retest reliability of the Stroop colour word test (Franzen et al., 1987)  Good performance validity (Erdodi et al., 2018) |
| Dual-task tests | Visual discrimination | Performing two simultaneous visual discrimination tasks, one with each hand. 3 conditions which are single- pure, single-mixed and dual-mixed (Esmail et al., 2020). | Esmail et al. (2020) study adapted the dual task tests from previous studies, therefore unable to report on psychometric properties of the exact version of the dual task tests used in the study. |
| Montreal cognitive assessment | Attention  Concentration  Executive functions Memory  Language  Visuo-constructional skills  Conceptual thinking Calculation  Orientation | A 30-item screening assessment tool used to detect mild cognitive impairment; each item assesses different areas of cognitive functioning (Nasreddine et al., 2005). | High sensitivity and specificity for detecting MCI, strong internal consistency, test-retest reliability and concurrent validity (Nasreddine et al., 2005). |
| Colour trails test  Trail making tests part A and B | Sustained and divided attention  Letter and number recognition  Mental flexibility  Visual scanning | Consists of 2 tasks:  1. Connecting consecutive numbers/circles (1-25) in ascending order.  2.Connecting ascending numbers/letters in alphabetical order in an alternating pattern (Mortimer et al., 2012; Anderson-Hanley et al., 2012; Sangkarat et al., 2017). | Colour trails test:  Strong test-retest reliability (D’Elia et al., 1996) and concurrent validity (Elkin-Frankston et al., 2007)  Trail making test:  Good test-retest reliability (Matarazzo et al., 1974)  Poor to adequate convergent validity (O’Donnell et al., 1994) |
| Digit span forward and backward test | Short term verbal memory  Working memory | Series of numbers called. Participants immediately repeat the same series of numbers in order and in inverse order (Anderson-Hanley et al., 2012; Monteiro-Junior et al., 2017). | Moderate criterion validity and  good test-retest reliability (Wechsler, 1997)  High internal reliability (Conway et al., 2005) |
| Frontal assessment battery | Executive functions | Brief tool encompassing 6 subtests of conceptualization, mental flexibility, motor programming, sensitivity to interference, inhibitory control and environmental autonomy (Dubois et al., 2000). | Good concurrent validity, inter-rater reliability, internal consistency, discriminant validity and sensitive to frontal lobe dysfunction (Dubois et al., 2000). |
| Mini mental state examination | Short term verbal memory  Construct ability  Visuo-spatial skills  Language  Calculation  Orientation  Attention | Screening test of 11 questions for detecting cognitive impairment. Each question relates to different areas of cognitive functioning (Folstein et al., 1975). | Adequate intra and inter-rater reliability (Molley & Standish, 1997).  Poor to excellent internal consistency and test-retest reliability (Tombaugh & McIntyre, 1992).  Excellent convergent validity (Snowden et al., 1999). |
| Touch M | Visuospatial function | Random display of blue colour signals divided into cells are presented. Participants reproduce the same arrangement of cells (Kitazawa et al., 2015). | High specificity and sensitivity in the diagnosis of Alzheimer’s disease, and good concurrent validity (Hatakeyama et al., 2006).  Limited evidence on the reliability of the Touch M. |
| Touch panel-type dementia assessment scale (TDAS) | Level of cognitive decline | Series of questions that test word recognition, following instructions, visuospatial perception, accuracy of the order of the process, naming fingers, money calculations, orientation, object recognition and clock time recognition (Kitazawa et al., 2015). | High concurrent validity and good sensitivity for detecting symptoms of Alzheimer’s disease (Inoue et al., 2011).  Limited evidence on the reliability of the TDAS. |
| Verbal fluency test  Category verbal fluency test  Category fluency test | Semantic, short term and working memory  Lexical access ability  Executive functioning | Participants name as many words in a particular category in a given time frame (Monteiro-Junior et al., 2017; Mortimer et al., 2012; Noice & Noice, 2013). | Strong validity and good reliability (Shao et al., 2014). |
| Mattis dementia rating scale | Attention  Initiation-perseveration Construction Conceptualisation  Memory | Series of questions/tasks testing attention, initiation-perseveration, construction, conceptualisation and memory (Foss et al., 2013). | High convergent and construct validity (Fernandez & Scheffel, 2003; Marson et al., 1997).  Good specificity and sensitivity for detecting cognitive impairment (Fernandez & Scheffel, 2003).  Mixed support for reliability (Smith et al., 1994). |
| Auditory verbal learning test | Verbal learning  Memory | First list of 15 words presented. Participants recall words from first list. Second list of 15 words presented. Participants recall words from 1^st^ and 2^nd^ lists (Schidt, 1996). | Adequate divergent and convergent validity (Magalhaes et al., 2012).  Good internal consistency (Magalhaes et al., 2012). |
| Word list recall (immediate and delayed) | Memory | 10 words belonging to different categories presented. Participants immediately and after 10mins recall the 10 words (Noice & Noice, 2013). | Good inter-rater, test-retest reliability and sensitivity to detecting dementia (Morris et al., 1989)  Limited evidence on validity. |
| East Boston memory test | Episodic memory  Verbal memory | Short paragraphs read to participants. Participants asked to immediately recall 12 elements of the story. Surprise delayed recall test after 4 mins (Noice & Noice, 2013). | Moderate concurrent validity and good sensitivity for measuring cognitive dysfunction (Gfeller & Horn, 1996)  Limited evidence on reliability. |
| Means-end problem solving procedure | Problem solving | 2 short stories presented. Each story has a ‘problem’ at the beginning and an ‘outcome’ at the end. Without limitations of time, participants asked to state events that might have happened in the middle of the story leading to the outcome (D’Zurilla & Maydeu-Olivares, 1995). | Adequate reliability and validity (D’Zurilla & Maydeu-Olivares, 1995). |
| Serial subtraction task | Complex attention  Processing speed | Counting backwards by threes whilst sitting (Silsupadol et al., 2009). | Adequate construct and discriminant validity of Serial 7s backwards task (Williams et al., 1996).  Limited empirical data on reliability (Karzmark, 2000). |
| Letter digit test | Processing speed | Matching digits and numbers as quickly as possible (Schoene et al., 2015). | Good validity and sensitive measure (Natu & Agarwal, 1995).  Limited evidence on reliability. |
| Simple hand and choice reaction time tests | Processing speed | Reacting to a random stimulus using a depression switch/stepping panel (Schoene et al., 2015; Sungkarat et al., 2016). | Good reliability (Lord et al., 2003) and concurrent validity (Buckhalt, 1991). |
| Attentional network test | Alerting  Orienting  Executive networks | A series of left/right pointing arrows presented with a central target in the middle of the screen. The central target is either a right or left pointing arrow. Participant identifies the direction of the target arrow (Fan et al., 2002). | Good reliability (Fan et al., 2002)  Good construct, criterion validity and test-retest reliability of the variant form of this test, Attention Network Test-Interaction test (Ishigami et al., 2016) |
| Mental rotation test | Visuo-spatial performance | 2 shapes presented. One of the shapes is clockwise and the other shape is anticlockwise. Participants identified whether the 2 shapes were identical or a mirror image of each other (Schoene et al., 2015). | Good internal consistency and test-retest reliability (Hirschfeld et al., 2013).  Adequate validity for computerised mental rotation tasks (Voyer et al., 2006). |
| Logical memory (delayed recall) subtest of Wechsler memory scale | Episodic memory | 2 stories orally presented. After 30 minutes, participants asked to repeat each story as accurately as possible (Sungkarat et al., 2016). | Strong internal consistency, test-retest reliability, construct and concurrent reliability and sensitive to change (Wechsler et al., 2009). |
| Block design test subtest of the Wechsler adult intelligence scale three | Visuospatial ability | Participants asked to copy a pattern using blocks. Number of blocks increased from four to nine (Sungkarat et al., 2016). | High validity and reliability (Ryan & Ward, 1999) |
| Lowenstein occupational therapy cognitive assessment- geriatric (LOTCA-G) | Orientation  Visual perception  Spatial perception  Praxis  Visuomotor organisation  Thinking operation  Memory | Consists of 23 subsets on:  place and time; object and shape identification; overlapping figures and object constancy; perception on examiner, self and surroundings; motor imitation; utilisation of objects and symbolic actions; drawing clock; reproduction of puzzle; block design; pegboard construction; two dimensional model and copying geometric forms; categorisation and pictorial sequencing; memory in identifying a famous personality; personal possession and everyday objects (Erez & Katz, 2004). | Good concurrent validity, internal consistency and  high inter-rater reliability (Katz et al., 1989)  Sensitive to detecting dementia (Erez & Katz, 2004). |
| Neuroplasticity assessment | Neuronal plasticity | BDNF levels via plasma samples analysed using enzyme-linked immunosorbent assay method (Anderson-Hanley et al., 2012; Bathina & Das., 2014). | Enzyme-linked immunosorbent assay is a valid and reliable method to determine BDNF levels (Elfving et al., 2010). |
| Brain scans | MRI scans to measure whole brain volume (Mortimer et al., 2012).    fNIRS scans (Eggenberger et al., 2016). | To calculate whole brain volume axial-oblique 3D fast spoiled gradient recalled echo (SPGR) sequence obtained. A detailed description of the specific MRI acquisition and analysis is described in Mortimer et al. (2012).  Functional near-infrared spectroscopy instrument (fNIRS) used to measure prefrontal cortex activity during walking (Eggenberger et al., 2016). | Good precision of measurements using magnetic resonance imaging to calculate whole brain volume (Steen et al., 2007). |

**Reference List**

Anderson-Hanley, C., Arciero, P.J., Brickman, A.M., Nimon, J.P., Okuma, N., Westen, S.C., Merz, M.E., Pence, B.D., Woods, J.A., Kramer, A.F., & Zimmerman, E.A. (2012). Exergaming and older adult cognition a cluster randomised clinical trial. *American Journal of Preventative Medicine, 42*(2), 109-119. <http://doi.org/10.1016/j.amepre.2011.10.016>

Bathina, S., & Das, U.N. (2014). Brain-derived neurotrophic factor and its clinical implications. *Archives of Medical Science, 11*(6), 1164-1178. <http://doi.org/10.5114/aoms.2015.56342>

Buckhalt, J.A. (1991). Reaction time measures of processing speed: Are they yielding new information about intelligence? *Individual difference, 12*(7), 683-688. <https://doi.org/10.1016/0191-8869(91)90223-X>

Conway, A., Kane, M., Bunting, M., Hambrick, Z., Wilhelm, O., & Engle, R. (2005). Working memory span tasks: A methodological review and user’s guide. *Psychonomic Bulletin and Review, 12*(5), 769-786. <https://link.springer.com/journal/13423/volumes-and-issues>

D’Elia, L. F., Satz, P., Uchiyama, C.L., & White, T. (1996). *Colour Trails Test.* PAR.

Dubois, B., Slachevsky, A., Litvan, I., & Pillon, B. (2000). The FAB a frontal assessment battery at bedside. *Neurology, 55*(11), 1621-1626. <http://doi.org/10.1212/wnl.55.11.1621>

D’Zurilla, T.J., & Maydeu-Olivares. (1995). Conceptual and methodological issues in social problem solving assessment. *Behaviour Therapy, 26*(3), 409-432. <https://doi.org/10.1016/S0005-7894(05)80091-7>

Eggenberger, P., Wolf, M., Schumann, M., & Bruin, E.D. (2016). Exergame and balance training modulate prefrontal brain activity during walking and enhance executive function in older adults, *Frontiers in Aging Neuroscience, 8*(66), 1-16. <http://doi.org/10.3389/fnagi.2016.00066>

Elfving, B., Plougmann, P.H., & Wegener, G. (2010). Detection of brain-derived neurotrophic factor (BDNF) in rat blood and brain preparations using ELISA: Pitfalls and solutions. *Journal of Neuroscience Methods, 187*(1), 73-77. <http://doi.org/10.1016/j.jneumeth.2009.12.017>

Elkin-Frankston, S., Lebowitz, B. K., Kapust, L. R., Hollis, A.M., & O’Connor, M.G. (2007). The use of the colour trails test in the assessment of driver competence: Preliminary reports of a culture-fair instrument. *Archives of Clinical Neuropsychology, 22*(5), 631-635. <https://doi.org/10.1016/j.acn.2007.04.004>

Erdodi, L.A., Sagar, S., Seke, K., Zuccato, B.G., Schwartz, E.S., & Roth, R.M. (2018). *Psychological Assessment, 30*(6), 755–766. <http://doi.org/10.1037/pas0000525>

Erez, A.B., & Katz, N. (2004). Cognitive profiles of individuals with dementia and heathy elderly: The Loewenstein occupational therapy cognitive assessment (LOTCA-G). *Physical & Occupational Therapy in Geriatrics, 22*(2), 29-42. <http://doi.org/10.1080/J148v22n02_03>

Esmail, A., Vrinceanu, T., Lussier, M., Predovan, D., Berryman, N., Houle, J., Karelis, A., Grenier, S., Vu, T.T., Villalpando, J.M., & Bherer, L. (2020). Effects of dance/movement training vs. aerobic exercise training on cognition, physical fitness and quality of life in older adults: A randomized controlled trial. *Journal of Bodywork & Movement Therapies, 24*(1), 212-220. <http://doi.org/10.1016/j.jbmt.2019.05.004>

Fan, J., McCandliss, B.D., Sommer, T., Raz, A., & Posner, M.I. (2002). Testing the efficiency and independence of attentional networks. *Journal of Cognitive Neuroscience, 14*(3), 340-347. <https://doi.org/10.1162/089892902317361886>

Fernandez, A.L., & Scheffel, D.L. (2009). Study of the criterion validity of the mattis rating scale. *International Journal of Testing, 3*(1), 49-58. <https://doi.org/10.1207/S15327574IJT0301_3>

Folstein, M. F., Folstein, S. E., & McHugh, P. R. (1975). “Mini-mental state”. A practical method for grading the cognitive state of patients for the clinician. *Journal of Psychiatric Research, 12*(3), 189-198. <http://doi.org/10.1016/0022-3956(75)90026-6>

Foss, M.P., Carvalho, V.A., Machado, T.H., Reis, G.C., Tumas, V., Caramelli, P., Nitrina, R., & Porto, C.S. (2013). Mattis dementia rating scale (DRS) normative data for the Brazilian middle-age and elderly populations. *Dementia Neuropsychology, 7(*4), 374-379. <http://doi.org/10.1590/S1980-57642013DN74000004>

Franzen, M.D., Tishelman, A.C., Sharp, B.H., & Friedman, A.G. (1987). An investigation of the test-retest reliability of the Stroop colour word test across two intervals. *Archives of Clinical Neuropsychology, 2*, 265-272. <https://academic.oup.com/acn>

Gajewski, P.D., Eva, H., Falkenstein, M., Thönes, S., & Wascher, E. (2018). What does the n-back task measure as we get older? Relations between working-memory measures and other cognitive functions across the lifespan. *Frontiers in Psychology, 9*, 1-17. <https://doi.org/10.3389/fpsyg.2018.02208>

Gfeller, J., & Horn, G. (1996). The East Boston Memory Test: A clinical screening measure for screening memory impairment in the elderly. *Journal of Clinical Psychology, 52*(2), 191-196. [http://doi.org/10.1002/(SICI)1097-4679(199603)52:2<191::AID-JCLP10>3.0.CO;2-F](http://doi.org/10.1002/(SICI)1097-4679(199603)52:2%3c191::AID-JCLP10%3e3.0.CO;2-F)

Hatakeyama, Y., Sasaki, R. Ikeda, N., Murakami, S., Nakano, N., & Saito, T. (2006). Newly developed task using touch screen device to estimate cognitive function especially visuospatial memory, executive function and executive and processing speed. *Psychogeriatrics, 17*(6), 655-664. <https://onlinelibrary.wiley.com/journal/14798301>

Hirschfeld, G., Thielsch, M.T., & Zernikow, B. (2013). Reliabilities of mental rotation tasks: Limits to the assessment of individual differences. *Biomedical Research International, 2013*, 1-7. <http://doi.org/10.1155/2013/340568>

Inoue, M., Jimbo, D., Taniguchi, M., & Urakami, K. (2011). Touch panel-type dementia assessment scale: A new computer-based rating scale for Alzheimer’s disease. *Psychogeriatrics, 11*(1), 28-33. <http://doi.org/10.1111/j.1479-8301.2010.00345.x>

Ishigami, Y., Eskes, G.A., Tyndall, A.V., Longman, R.S., Drogos, L.L., & Poulin, M.J. (2016). The attention network test-interaction (ANT-I): Reliability and validity in healthy older adults. *Experimental Brain Research, 234*(3), 815-827. <https://doi.org/10.1007/s00221-015-4493-4>

Jaeggi, S.M., Buschkuehl, M., Perrig, W.J., & Meier, B. (2010). The concurrent validity of the n-back task as a working memory measure. *Memory, 18*(4), 394-412. <https://doi.org/10.1080/09658211003702171>

Karzmark, P. (2000). Validity of the serial seven procedure. *International Journal of Geriatric Psychiatry, 15*(8), 677-679. [https://doi.org/10.1002/1099-1166(200008)15:8<677::AID-GPS177>3.0.CO;2-4](https://doi.org/10.1002/1099-1166(200008)15:8%3c677::AID-GPS177%3e3.0.CO;2-4)

Katz, N., Itzkovich, M., Averbuch, S., & Elazar, B. (1989). Loewenstein occupational therapy cognitive assessment (LOTCA) battery for brain-injured patients: Reliability and validity. *American Journal of Occupational Therapy, 43*(3), 184-192. <http://doi.org/10.5014/ajot.43.3.184>

Kearney-Ramos, T.E., Fausett, J.S., Gess, J.L., Reno, A., Peraza, J., Kilts, C.D., & James, G.A. (2014). Merging clinical neuropsychology and functional neuroimaging to evaluate the construct validity and neural network engagement of the n-back task. *Journal of the International Neuropsychological Society, 20*, 736–750. <https://doi.org/10.1017/S135561771400054X>

Kitazawa, K., Showa, S., Hiraoka, A., Fushiki, Y., Sakauchi, H., & Mori, M. (2015). Effect of a dual task net step exercise on cognitive and gait function in older adults. *Journal of Physical Therapy, 38*(3), 133-140. <http://doi.org/10.1519/JPT.0000000000000029>

Kulikowski, K., & Potasz-Kulikowska. K. (2016). Can we measure working memory via the Internet? The reliability and factorial validity of an online n-back task. *Polish Psychological Bulletin, 47*(1) 51–61. <https://doi.org/10.1515/ppb-2016-0006>

Lord, S.R., Menz, H.B., & Tiedemann, A. (2003). A physiological profile approach to falls risk assessment and prevention. *Physical Therapy, 83*(3), 237-252. <https://doi.org/10.1093/ptj/83.3.237>

Magalhaes, S.S., Hamdan, A., & Malloy-Diniz, L.F. (2012). Validity convergent and reliability test-retest of the Rey Auditory Verbal Learning Test. *Archives of Clinical Neuropsychology, 9*(3), 129-137. <https://academic.oup.com/acn>

Marson, D.C., Dymek, M.P., Duke, L.W., & Harrell, L.E. (1997). Subscale validity of the mattis dementia rating scale. *Archives of Clinical Neuropsychology, 12*(3), 269-275. <https://doi.org/10.1016/S0887-6177(96)00003-0>

Matarazzo, J.D., Wiens, A.N., Matarazzo, R.G., & Goldstein, S.G. (1974). Psychometric and clinical test-retest reliability of the Halstead Impairment Index in a sample of healthy, young, normal men. *The Journal of Nervous and Mental Disease, 188*(1), 37-49. <http://doi.org/10.1097/00005053-197401000-00006>

Molloy, D. W., & Standish, T. I. M. (1997). A guide to the standardized Mini-Mental State Examination. *International Psychogeriatrics, 9*(1), 87-94. <https://doi.org/10.1017/S1041610297004754>

Moneiro-Junior, R.S., Figueiredo, L.F., Maciel-Pinheiro, P.T., Adub, E.L., Braga, A.E., Barca, M.L., Engedal, K., Nascimento, O.J., Deslandes, A.C., & Laks, J. (2017). Acute effects of exergames on cognitive function of institutionalised older persons: A single blinded, randomized and controlled pilot study. *Aging Clinical Experimental Research, 26*, 387-394. <http://doi.org/10.1007/s40520-016-0595-5>

Morris, J.C., Heyman, A., Mohs, R.C., Hughes, J.P., Belle, G.V., Fillenbaum, G., Mellits, E.D., & Clark, C. (1989). The consortium to establish a registry for Alzheimer’s Disease (CERAD). Part I. Clinical and neuropsychological assessment of Alzheimer’s disease. *Neurology, 39*(9), 1159-1195. <http://doi.org/10.1212/wnl.39.9.1159>

Mortimer, J.A., Ding, D., Borenstein, A.R., DeCarli, C., Guo, Q., Wu, Y., Zhao, Q., & Chu, S. (2012). Changes in brain volume and cognition in a randomised trial of exercise and social interaction in a community-based sample of non-demented Chinese elders. *Journal of Alzheimer’s of Disease, 30*(4), 757-766. <http://doi.org/10.3233/JAD-2012-120079>

Nasreddine, Z. S., Phillips, N. A., Bediriam, V., Charbonneau, S., Whitehead, V., Collin, I., Cummings, J. L., & Chertkow, H. (2005). The Montreal cognitive assessment, Moca: A brief screening tool for mild cognitive impairment. *Journal of the American Geriatrics Society, 53*(4), 695-699. <https://doi.org/10.1111/j.1532-5415.2005.53221.x>

Natu, M.V., & Agarwal, A.K. (1995). Digit letter substitution (DLST) as an alternative to digit symbol substitution test (DSST). *Human Psychopharmacology, 10*(4)*,* 339-343. <https://doi.org/10.1002/hup.470100414>

Noice, H., & Noice, T. (2013). Extending the reach of evidence-based theatrical intervention. *Experimental Aging Research, 39*, 398-418. <http://doi.org/10.1080/0361073X.2013.808116>

Norouzi, E., Vaezmosavi, M., Gerber, M., Puhse, U., & Brand, S. (2019). Dual-task training on cognition and resistance training improved both balance and working memory in older people. *The Physician and Sportsmedicine,* *47*(4), 471-478. <https://doi.org/10.1080/00913847.2019.1623996>

O’Donnell, J.P., Macgregor, L.A., Dabrowski, J.J., Oestreicher, J.M., & Romero, J.J. (1994). Construct validity of neuropsychological tests of conceptual and attentional abilities. *Journal of Clinical Psychology, 50(*4), 596-560. [http://doi.org/10.1002/1097-4679(199407)50:4<596::aid-jclp2270500416>3.0.co;2-s](http://doi.org/10.1002/1097-4679(199407)50:4%3c596::aid-jclp2270500416%3e3.0.co;2-s)

Ryan, J.J., & Ward, L.C. (1999). Validity, reliability and standard errors of measurement for two seven subtest short forms of the wechsler adult intelligence scale III. *Psychological Assessment, 11*(2), 207-211. <https://doi.org/10.1037/1040-3590.11.2.207>

Schmidt, M. (1996). *Rey Auditory and Verbal Learning Test*. Western Psychological Association.

Schoene, D., Valenzuela, T., Toson, B., Delbaere, K., Severino, C., Garcia, J., Davies, T.A., Russell, F., Smith, S.T., & Lord, S.R. (2015). Interactive cognitive-motor step training improves cognitive risk factors of falling in older adults: A randomised trial. *PLOS ONE, 10*(12), 1-18. <http://doi.org/10.1371/journal.pone.0145161>

Shao, Z., Janse, E., Visser, K., & Meyer, A.S. (2014). What do verbal fluency tasks measure? Predictors of verbal fluency performance in older adults. *Frontiers in Psychology, 5*, 1-10. <http://doi.org/10.3389/fpsyg.2014.00772>

Silsupadol, P., Lugade, V., Shumway-Cook, A., Donkelaar, P.V., Chou, L.S., Mayr, U., Woollacott, M.H. (2009). Training-related changes in dual-task walking performance of elderly persons with balance impairment: A double blind, randomised controlled trial. *Gait Posture, 29*(4), 634-639. <http://doi.org/10.1016/j.gaitpost.2009.01.006>

Smith, G.E., Ivnik, R.J., Malec, J.F., Kokmen, E., Tangalos, E., & Petersen, R.C. (1994). Psychometric properties of the mattis dementia rating scale. *Assessment, 1*(2), 123-131. <http://doi.org/10.1177/1073191194001002002>

Snowden, M., McCormick, W., Russo, J., Srebnik, D., Comtois, K., Bowen, J., Teri, L., & Larson, E. B. (1999). Validity and responsiveness of the minimum data set. *Journal of the American Geriatrics Society, 47*(8), 1000-1004. <https://doi.org/10.1111/j.1532-5415.1999.tb01297.x>

Steen, R.G., Hamer, R.M., & Lieberman, J.A. (2007). Measuring brain volume by MR imaging: Impact of measurement precision and natural variation on sample size requirements. *American Journal of Neuroradiology, 28*(6), 1119-1125. <https://doi.org/10.3174/ajnr.A0537>

Sungkarat, S., Boripuntakul, S., Chattipakorn, N., Watcharasaksilp, K., & Lord, S.R. (2017). Effects of tai chi on cognition and fall risk in older adults with mild cognitive impairment: A randomised controlled trial. *Journal of the American Geriatrics Society, 65*(4), 721-727. <http://doi.org/10.1111/jgs.14594>

Tombaugh, T. N., & McIntyre, N. J. (1992). The Mini-Mental State Examination: A comprehensive review. *Journal of the American Geriatrics Society, 40*(9), 922-935. <http://doi.org/10.1111/j.1532-5415.1992.tb01992.x>

Voyer, D., Butler, T., Cordero, J., Brake, B., Silbersweig, D., Stern, E., & Imperato-McGinley, J. (2006). The relation between computerised and paper and pencil mental rotation tasks: A validation study. *Journal of Experimental Neuropsychology, 28*(6), 928-939. <http://doi.org/10.1080/13803390591004310>

Wechsler, D., Holdnack, J. A., & Drozdick, L. W. (Ed.). (2009). *Wechsler memory scale: Technical and interpretive manual*. Pearson.

Wechsler, D. (1997). *Manual for the Wechsler Adult Intelligence scale-III*. Psychological Corporation.

Wechsler, D. (1997). *WAIS III administration and scoring manual.* The Psychological Corporation.

Williams, M.A., LaMarche, J.A., Alexander, R.W., Stanford, L.D., Fielstein, E.M., & Boll, T.J. (1996). Serial 7s and alphabet backwards as brief measures of information processing speed. *Archives of Clinical Neuropsychology, 11*(8), 651-659. <https://academic.oup.com/acn>
